# High rate of occult cancer found in prophylactic mastectomy specimens despite thorough presurgical assessment with MRI and ultrasound: findings from the Hereditary Breast and Ovarian Cancer Registration 2016 in Japan

**DOI:** 10.1007/s10549-018-4953-1

**Published:** 2018-09-10

**Authors:** Hideko Yamauchi, Megumi Okawa, Shiro Yokoyama, Chizuko Nakagawa, Reiko Yoshida, Koyu Suzuki, Seigo Nakamura, Masami Arai

**Affiliations:** 1 Department of Breast Surgical Oncology, St. Luke’s International Hospital, 9-1 Akashi-cho, Chuo-ku, Tokyo, 104-8560 Japan; 2 The Japanese HBOC Consortium, Division of Breast Surgical Oncology, Department of Surgery, Showa University School of Medicine, 1-5-8 Hatanodai, Shinagawa-ku, Tokyo, 142-8666 Japan; 3 Clinical Genetic Oncology, Cancer Institute Hospital of Japanese Foundation for Cancer Research, 3-8-31, Ariake, Koto-ku, Tokyo, 135-8550 Japan; 4 Department of Pathology, St. Luke’s International Hospital, 9-1 Akashi-cho, Chuo-ku, Tokyo, 104-8560 Japan; 5 Division of Breast Surgical Oncology, Department of Surgery, Showa University School of Medicine, 1-5-8 Hatanodai, Shinagawa-ku, Tokyo, 142-8666 Japan; 6 Diagnostics and Therapeutics of Intractable Disease, Juntendo University, Graduate School of Medicine, 2-1-1 Hongo, Bunkyo-ku, Tokyo, 113-8421 Japan

**Keywords:** BRCA, Hereditary breast and ovarian cancer syndrome, Magnetic resonance imaging, Occult cancer, Pathological method, Prophylactic mastectomy

## Abstract

**Purpose:**

Prophylactic surgery is a preemptive strategy for hereditary breast and ovarian cancer (HBOC). Prophylactic mastectomy (PM) reduces breast cancer risk by > 90%. The aim of our study is to analyze the information of the Japanese pedigrees and to utilize the results for clinical practice.

**Methods:**

We statistically analyzed records of HBOC registrees who had undergone *BRCA1*/*2* genetic testing at seven medical institutions up until 2016. In the cases of PM, we examined breasts with the use of mammography (MMG), ultrasound (US), and magnetic resonance imaging (MRI) before surgery. After PM, the specimens were divided about 1 cm serially and examined in their entirety.

**Results:**

Of 1527 registrees who underwent *BRCA* testing, 1125 (73.7%) were negative for *BRCA1*/*2* mutation, 297 (19.5%) were positive for *BRCA1*/*2* mutation (*BRCA1*/*2*^*MUT*+^), and 105 (6.9%) had uncertain results. To decide whether to undergo total mastectomy vs. breast-conserving surgery (BCS), 370 registrees underwent presurgical genetic testing. During the follow-up period, four new-onset breast cancers were found among the 55 non-affected BRCA carriers. Among the 73 *BRCA1*/*2*^*MUT*+^ carriers who underwent BCS, 3 were found to have ipsilateral breast cancer. Of 189 *BRCA1*/*2*^*MUT*+^ carriers with unilateral breast cancer, 8 were found to have contralateral breast cancer. Of 53 PM specimens, 6 (11.3%) were found to have occult breast cancer despite using MMG, US, and MRI.

**Conclusions:**

Our report showed a relatively higher incidence rate of occult cancer at 11.3% in PM specimens despite thorough pre-operative radiological evaluations, which included a breast MRI. Considering the occult cancer rates and the various pathological methods of our study and published studies, we propose the necessity of a histopathological protocol.

## Introduction

The breast cancer rate tends to peak at a younger range in Japan than in Western countries [1–3]. About half of breast cancer diagnoses in Japan are for patients in their 30s–50s [2]. As this pattern probably reflects their genetic background, investigations of hereditary breast and ovarian cancer (HBOC) are important for Japanese women. The Japanese nationwide HBOC registration system aims to clarify clinical and genetic features of Japanese HBOC and to improve its medical treatment.

The Japanese HBOC Consortium (JHC) was established in December 2012. We established a registration committee for JHC in October 2013 and promoted it as a nationwide registration project. The registered subjects were all Japanese individuals who underwent *BRCA1*/*2* genetic testing (including individuals in which no mutation was detected) [4]. Here, we report results of the HBOC Registration from its establishment until 2016. The objective of the current study is to analyze the information of the Japanese pedigrees, who underwent *BRCA1*/*2* genetic testing, and to make use of the results in clinical practice.

Prophylactic surgery, such as prophylactic mastectomy (PM), is a preemptive strategy for HBOC. As PM can reduce risk of breast cancer by > 90%, it is often performed among *BRCA1*/*2* mutation (*BRCA1*/*2*^*MUT*^) carriers. Reportedly, occult cancers are detected in 0.5–9.9% of PM specimens [5–15].

## Methods

This study included subjects who underwent *BRCA1*/*2* genetic testing until 2016. As of 2016, 7 participating medical institutions were enrolled: St. Luke’s International Hospital (Tokyo), Cancer Institute Hospital (Tokyo), Showa University Hospital (Tokyo), Hoshi General Hospital (Fukushima), Kitano Hospital (Osaka), Shikoku Cancer Center (Ehime), and Kochi Medical School Hospital (Kochi).

All subjects, who received genetic counseling and underwent genetic testing of their own free will in clinical practice, were those who had been provided explanations of the HBOC risk in accordance with Genetic/Familial High-Risk Assessment: Breast and Ovarian in NCCN Guidelines [16]. Most of genetic testing with sequencing and large rearrangement analysis was performed at Myriad Genetic Laboratories or FALCO Biosystems. Detected variants were interpreted by the criteria of Myriad Genetic Laboratories. We entered information for *BRCA1*/*2* genetic testing and clinicopathological findings of breast cancer, ovarian cancer, and other cancers in the original electronic template. All data except sex were anonymously registered in each institution. Dates of birth only included year and month [4].

In the cases of PM, we examined breasts with the use of mammography (MMG), ultrasound (US), and magnetic resonance imaging (MRI) before surgery. After PM, the specimens were processed by a pathologist. Although a surgicopathological protocol for occult cancer in the PM specimens does not exist, the specimens were divided about 1 cm serially and examined in their entirety.

## Results

**Fig. 1:**
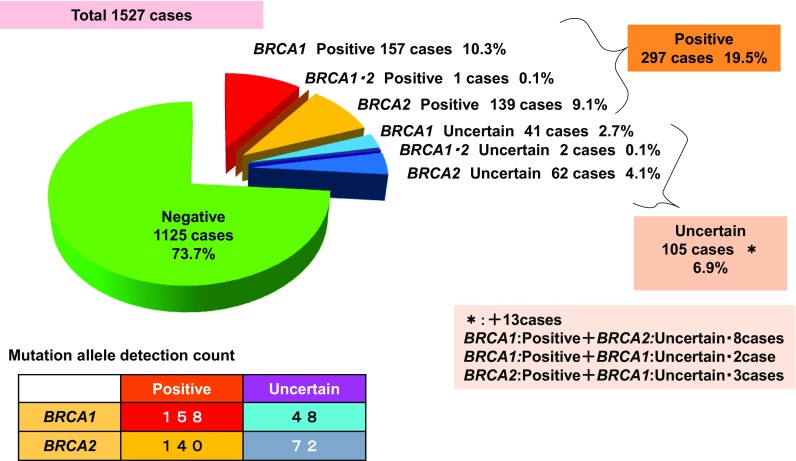
Prevalence of *BRCA1*/*2* mutations

**Fig. 2:**
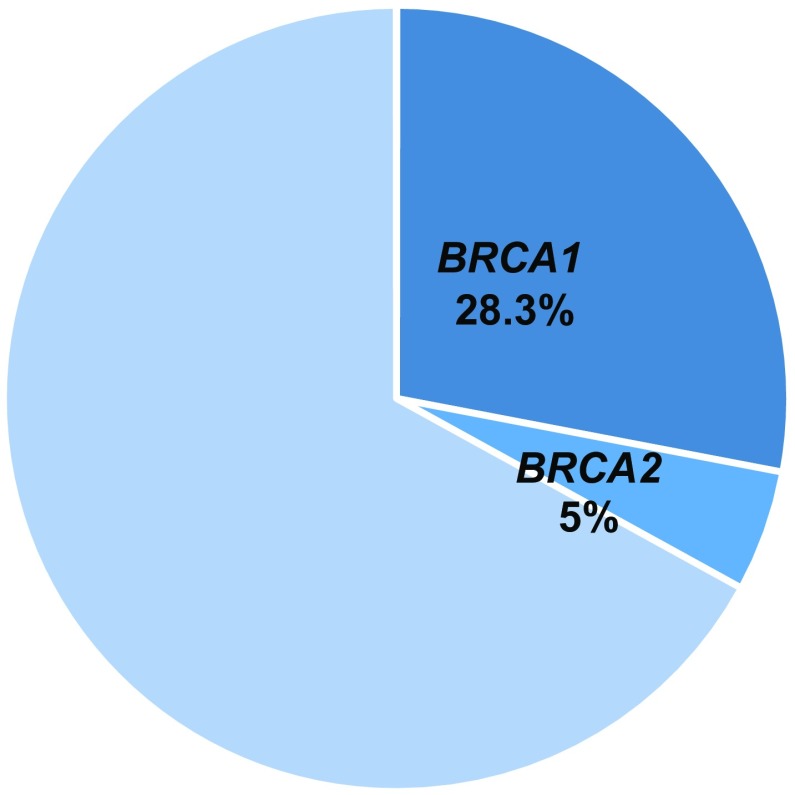
Rates of *BRCA1*/*2* mutations in triple-negative breast cancers

**Fig. 3:**
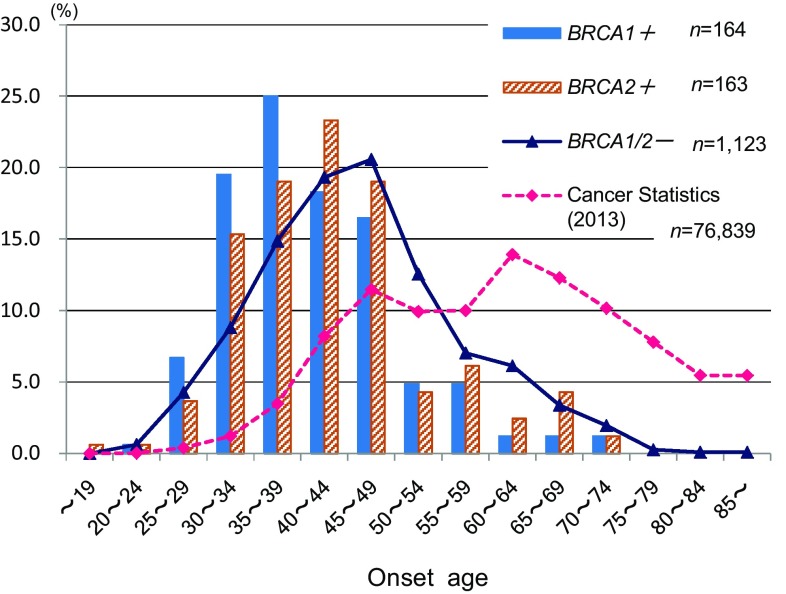
Distribution of age at onset of breast cancer with/without *BRCA1*/*2* mutations and national statistics (2013). Mean age at onset of breast cancer. *BRCA1*/*2* mutation positive: 41.7 years; *BRCA1*/*2* mutation negative: 45.8 years

Of 1527 registrees who underwent *BRCA* testing, 1125 (73.7%) were negative for *BRCA1*/*2* mutation (*BRCA1*/*2*^*MUT*−^), 297 (19.5%) were positive for *BRCA1*/*2* mutation (*BRCA1*/*2*^*MUT*+^), and 105 (6.9%) had uncertain results. Among the 297 *BRCA1*/*2*^*MUT*+^ subjects (19.5%), 157 (10.3%) carried mutations for *BRCA1*, 139 (9.1%) for *BRCA2*, and 1 (0.1%) was positive for both (Fig. 1). Among 359 patients with triple-negative breast cancer, 101 (28.3%) had mutations for *BRCA1* and 18 (5.0%) for *BRCA2* (Fig. 2). Distribution of age at onset of breast cancer with/without *BRCA1*/*2* mutations (Fig. 3) shows that *BRCA1*/*2*^*MUT*+^ breast cancer occurred at a younger mean age (41.7 years) than did *BRCA1*/*2*^*MUT*−^ breast cancer (45.8 years). In comparison to the 2013 National Registration for Breast Cancer Incidence in Japan (*n* = 76,839) [2], breast cancer with *BRCA* mutations occurred at a younger age. Among types of *BRCA1*/*2* pathological mutations that were reported more than once, L63X was the most common (Table 1).

**Table 1:** Types of *BRCA1*/*2* pathological mutations that were reported more than once

*BRCA1*				*BRCA2*			
Base mutation		Amino acid mutation	Report count	Base mutation		Amino acid mutation	Report count
Myriad	HGVS			Myriad	HGVS		
307T > A	c.188T > A	L63X	40	5804del4	c.5576_5579delTTAA	STOP1862	10
Unconfirmed			1	7180C > T	c.6952C > T	R2318X	9
2919C > T	c.2800C > T	Q934X	13	8732C > A	c.8504C > A	S2835X	7
3561delG	c.3442delG	STOP1154	5	Unconfirmed			1
575delCA	c.456_457delCA	STOP157	5	9345G > A	c.9117G > A	P3039P	6
2508delGA	c.2389_2390delGA	STOP799	4	1506delA	c.1278delA	STOP429	6
3759G > T	c.3640G > T	E1214X	4	8857G > T	c.8629G > T	E2877X	5
5083C > T^b^	c.4964C > T	S1655F	4	9304C > T	c.9076C > T	Q3026X	5
IVS20-1G > A	c.5278-1G > A		3	5873C > A	c.5645C > A	S1882X	5
IVS20-1G > C	c.5278-1G > C		3	8817insA	c.8589dupA	STOP2868	5
1623del5	c.1504_1508delTTAAA	STOP503	2	2041delA	c.1813dupA	STOP613	5
297C > T	c.178C > T	Q60X	2	5804del4	c.5576_5579delTTAA	STOP1861	4
309T > C	c.190T > C	C64R	2	9610C > T	c.9382C > T	R3128X	3
5181del3	c.5062delGTT	V1688del	2	3463delT	c.3235delT	STOP1086	3
5280C > T	c.5161C > T	Q1721X	2	983del4	c.755_758delACAG	STOP275	3
exon1a-2del	c.(?_-1387-1)_(80 + 1_81 − 1)del		2	4123G > T	c.3895G > T	E1299X	2
exon8 del	c.(441 + 1_442-1)_(546 + 1_547-1)del		2	8251A > G^b^	c.8023A > G	I2675V	2
IVS14-2A > G^a^	c.4485-2A > G		2	3423del4	c.3195_3198delTAAT	STOP1075	2
				2041insA	c.1813dupA	STOP615	2
				3036del4	c.2808_2811delACAA	STOP959	2

^a^Suspected deleterious

^b^Mixed suspected deleterious and deleterious

To help decide between surgical procedures (total mastectomy vs. breast-conserving surgery [BCS]), 370 subjects underwent presurgical genetic testing. Of the 66 *BRCA1*/*2*^*MUT*+^ subjects, 58 (87.9%) chose to undergo total mastectomy, and 8 (12.1%) chose BCS. Of the 304 *BRCA1*/*2*^*MUT*−^ subjects, 141 (46.4%) chose total mastectomy, 158 (52.0%) chose BCS, and 5 had unknown choices (Table 2).

**Table 2:** Genetic testing to select breast cancer surgical procedures (*n* = 418)

Testing results	Cases count	Breast cancer operation type		
		Breast-conserving surgery	Mastectomy	Unknown
Positive	66	8 12.1%	58 87.9%	0 0%
Negative	304	158 52.0%	141 46.4%	5 1.6%

# of 418 patients, 370 underwent surgery after genetic testing

During the follow-up period, four cases of new-onset breast cancers were observed among the 55 non-affected BRCA carriers (mean observation period: 2.5 years; incidence rate: 2.9%/year; Table 3). Among the 73 *BRCA1*/*2*^*MUT*+^ women who underwent BCS, 3 ipsilateral breast cancer cases were found (mean observation period: 3.5 years; incidence rate: 1.2%/year), compared with only 2 cases among the 477 *BRCA1*/*2*^*MUT*−^ women (mean observation period: 2.2 years; incidence rate: 0.2%/year; Table 4). Of 189 *BRCA1*/*2*^*MUT*+^ women with unilateral breast cancer, 8 contralateral breast cancer cases were found (mean observation period: 3.0 years; incidence rate: 1.4%/year), compared with 4 cases of contralateral breast cancer among 892 *BRCA1*/*2*^*MUT*−^ women (mean observation period: 2.2 years; incidence rate: 0.2%/year; Table 5).

**Table 3:** Breast cancer after genetic testing among non-affected *BRCA* carriers

Carriers without a history of breast cancer^a^	55 cases
Observation period after genetic testing (average)	0–13.9 years (2.5)
Age at genetic testing (average)	Age 20–66 (age 38.6)
Breast cancer onset after genetic testing	4 cases
Incidence rate	4/137.5 (persons/person years) 2.9%/year
***BRCA1*** positive 2 cases, ***BRCA2*** positive 2 cases	
Opportunities for detection	MRI: 2 cases, DCIS MMG: 1 case, DCIS Self-detection: 1 case, invasive 3.2 cm

*DCIS* ductal carcinoma in situ, *MMG* mammography, *MRI* magnetic resonance imaging

^a^Including one patient with a history of cervical cancer and another with a history of ureter cancer

**Table 4:** Breast cancers in ipsilateral breasts after breast-conserving surgery

	*BRCA1*/*2* positive	*BRCA1*/*2* negative
Women with a history of breast-conserving surgery	73 cases	477 cases
Ipsilateral breast cancer onset after genetic testing	3 cases	2 cases
Observation period after genetic testing (average)	0.01–12.3 years (3.5)	0.01–12.5 years (2.2)
Incidence rate	3/256 (persons/person years) 1.2%/year	2/1049 (persons/person years) 0.2%/year
Background		
Age of onset of the first breast cancer (average)	Age 19–71 (age 41.7)	Age 22–81 (age 46.4)
Number of exclusion cases due to PM	3 cases	0 cases

*PM* prophylactic mastectomy

**Table 5:** Contralateral breast cancers among patients treated for unilateral breast cancers

	*BRCA1*/*2* positive	*BRCA1*/*2* negative
Women with a history of unilateral breast cancer	189 cases	892 cases
Contralateral breast cancer onset after genetic testing	8 cases	4 cases
Observation period after genetic testing (average)	0.02–16.8 years (3.0)	0.01–20.2 years (2.2)
Incidence rate	8/567 (persons/person years) 1.4%/year	4/1962 (persons/person years) 0.2%/year
Background		
Age of onset of the first breast cancer (average)	Age 19–74 (age 41.7)	Age 22–85 (age 45.4)
Number of exclusion cases due to PM	37 cases	3 cases

*PM* prophylactic mastectomy

Among the 51 patients who underwent PM (Table 6), 6 had specimens in which occult breast cancer was found, including 1 with a *BRCA1* mutation and 5 with *BRCA2* mutations. All six patients had undergone extensive imaging prior to PM, using MMG, US, and breast MRI (Tables 7, 8). In our study, the rate of occult cancer among total removed breasts by PM was 6/53 = 11.3%.

**Table 6:** Clinicopathological characteristics of patients who underwent prophylactic mastectomies (*n* = 51)

Age	
Mean	43.7
Median	43
Range	30–62
*BRCA1*	
Positive	29 cases
*BRCA2*	
Positive	18 cases
*BRCA1*/*2*	
Negative	4 cases
Breast cancer stage	
0	3 cases
1	17 cases
2	14 cases
3	4 cases
4	0 case
Non-onset	2 cases
Uncertain	11 cases
Breast cancer subtype	
Hormone positive	
HER2 negative	11 cases
HER2 positive	3 cases
HER2 uncertain	8 cases
Hormone negative	
HER2 positive	0 case
HER2 negative	23 cases
HER2 uncertain	2 cases
Non-onset	2 cases
Uncertain	2 cases

*HER2* human epidermal growth factor receptor 2

**Table 7:** Clinicopathological characteristics of patients in whom occult cancer was found after undergoing prohylactic mastectomies

Age	
Mean	42.2
Median	43
Range	33–51
*BRCA1*	1 case
*BRCA2*	5 cases
Occult cancer	
DCIS	5 cases
Invasive cancer	1 case

*DCIS* ductal carcinoma in situ

**Table 8:** Cases of occult cancer in this study

	Age	*BRCA1* or *2*	Size (cm)	Type
1	36	2	NA	DCIS
2	47	2	NA	DCIS
3	43	1	NA	DCIS
4	51	2	0.5	Invasive
5	43	2	NA	DCIS
6	33	2	NA	DCIS

*DCIS* ductal carcinoma in situ, *NA* not available

## Discussions

We report herein one of the highest incidence rates in the literature: 11.3% of occult cancer in PM specimens, despite thorough presurgical assessment with MRI, US, and MMG, compared with previously reported rates of 0.5–9.9% (Table 9). We reviewed several factors thought to influence occult cancer occurrence, including (a) rates of bilateral prophylactic mastectomy (BPM), (b) pre-PM examination methods, (c) *BRCA1*/*2*^*MUT*+^ rates among subjects, and (d) pathological methods.

**Table 9:** Occult cancers reported in the literature

References	Subjects#	% of BRCA	# of BPM	# of Total PM	Occult cancer rate by total PM#	Pre-PM exam	Pathological method
Hartmann [5]	645	NA	645	1290	6/1290 (0.5%)	NA	NA
Meijers-Heijboer [6]	76	100	76	152	1/152 (0.7%) LCIS:1, No DCIS or IDC	PE, MMG, or MRI	3 random blocks/quadrant
Yao [7]	150	100	148	298	4/298 (1.3%) IDC:1, DCIS:3	PE, MMG, or US, All MRI	NA
Burger [8]	71	8.5	12	83	4/83 (4.8%) ILC(3.5 mm):1, LCIS:3	NA	NA
Boughey [9]	409	5.6	27	436	22/436 (5.0%) IDC:2, ILC:6 (IDC&ILC:2–9 mm) DCIS:14	PE, MMG	2 section/each quadrant & nipple
van Sprundel [10]	79	100	0	79	4/79 (5.1%) IDC(32 mm):1, DCIS:3	PE, radiological	NA
McLaughlin [11]	529	9.3	84	613	33/613 (5.4%) IDC:10, DCIS:23	PE, MMG, (US and/or MRI), (235/529pts: MRI)	2 section/each quadrant & nipple
Evans [12]	105	100	0	105	6/105 (5.7%) IDC:4, DCIS:2	NA	NA
Hoogerbrugge [13]	67	66	41	108	10/108 (9.3%) IDC(4 mm):1 DCIS(2–40 mm):9 (17/67pts: LCIS)^a^	PE, MMG, 4/10pts MRI, (27/67pts: MRI)	5 mm slices and radiological exam, then suspicious lesions and randomly selected each quadrant and nipple (Ave. 19 slides)
Kauff [14]	24	100	7	31	3/31 (9.7%) DCIS(7–20 mm):3 (LCIS: 1)^a^	MMG	2–4 section/each quadrant& nipple
Black [15]	173	17	19	192	19/192 (9.9%) IDC(1.5–10 mm):5, DCIS:14	59/173pts MRI	NA
Our study	51	92	2	53	6/53 (11.3%) IDC(5 mm):1, DCIS:5	PE, MMG, US & MRI	About 1 cm slices

*BPM* bilateral prophylactic mastectomy, *DCIS* ductal carcinoma in situ, *IDC* invasive ductal carcinoma, *ILC* invasive lobular carcinoma, *LCIS* lobular carcinoma in situ, *MMG* mammography, *MRI* magnetic resonance imaging, *NA* not available, *PE* physical examination, *PM* prophylactic mastectomy, *US* ultrasound

^a^LCIS were detected, but not included, as occult cancer cases, as they may co-exist with DCIS

### Rates of BPM

Rates of BPM among subjects in the first three studies of Table 9 are higher (at or near 100%) than in the other studies. The retrospective study of Hartmann et al. [5] included all women with family histories of breast cancer who underwent BPM in USA between 1960 and 1993. They found only 0.5% of occult cancer after BPM, though the rate of *BRCA* mutations among their subjects was not available. Meijers-Heijboer et al. [6] conducted a prospective study of 139 women with pathogenic *BRCA1* or *BRCA2* mutations who were enrolled in a breast-cancer surveillance program, Netherlands. Of the 139, 76 underwent PM from 1992 to 2001. They found only 1 case of lobular carcinoma in situ [LCIS (0.7%)] and no cases of ductal carcinoma in situ (DCIS) or invasive ductal carcinoma (IDC), even among *BRCA1*/*2*^*MUT*^ carriers. The study of Yao et al. [7] was a retrospective review of pathology results and outcomes of 201 *BRCA1*/*2*^*MUT*^ carriers, in USA, treated between 2007 and 2014 (1.3% occult cancer among 150 *BRCA1*/*2*^*MUT*^ carriers [298 breasts] undergoing nipple-sparing PMs). The much higher rates of BPM in these three studies seem to have resulted in much lower occult cancer rates (0.5–1.3%) than in the other studies. BPM patients are considered to have no history of breast cancer.

In contrast, van Sprundel et al. [10] in the Netherlands found 5% occult cancer among 79 of 148 patients who underwent contralateral prophylactic mastectomy (CPM). The 148 patients were identified until June 2003 as carrying *BRCA1* or *BRCA2* mutations with previous histories of unilateral, stage I–IIIa invasive breast cancer. Evans et al. [12] in UK between 1985 and 2010, considered whether CPM improves overall survival, and found 5.7% occult cancer in 105 women with *BRCA1*/*2* mutations and unilateral breast cancer who underwent CPM. By comparing the 100% (or near-100%) BPM cohorts with 100% CPM cohorts, we see that high rates of BPM might be associated with lower rates of occult cancer. Therefore, even among *BRCA* carriers, detection rate of occult cancer may have been influenced by the status whether affected or non-affected.

### Pre-PM examination methods

Black et al. [15] of USA reviewed occult malignancy in 192 PMs in 173 patients treated from 1999 to 2005, to compare pre-operative MRI with sentinel lymph node biopsy (SLNB), and found that MRI (performed in 59 patients) missed three of four total occult cancers. In the study of McLaughlin et al. [11] (USA) of 529 patients who underwent 613 PMs between 1999 and 2006, both pre-operative MRI and SLNB were performed selectively at the discretion of the surgeon; they reported the sensitivity of MRI for detecting occult cancers to be 78%. In a 2015 study, Riedl et al. [17] insisted that the use of MRI to screen women at increased risk for breast cancer improved detection of invasive cancers and DCIS, regardless of mutation status, age, or breast density; their improved results for MRI sensitivity might be explained by technical advances, improved diagnostic criteria, and greater familiarity of radiologists in reading breast MRIs, including the ability to diagnose DCIS with MRI [17, 18].

Regarding US, Bosse et al. [19] reported with respect to *BRCA1*/*2*^*MUT*^ carriers, that the sensitivity of US was 77%, and that of MRI was 100%. Ohuchi et al. [20] from Japan reported that the sensitivity of MMG + US for asymptomatic women aged 40–49 years with no history of any cancer in the previous 5 years was 91.1%.

With regard to MRI + MMG + US (yearly MRI, MMG, and biannual US), van Zelst et al. [21] reported the sensitivity to be 76.3% for surveillance of *BRCA1*/*2*^*Mut*+^ women and their first-degree untested relatives. Riedl et al. [17] reported the sensitivity to be 95.0%, among *BRCA1*/*2*^*Mut*+^ carriers and women with a familial risk > 20% (US was offered to *BRCA* mutation carriers). Kuhl et al. [22] reported the sensitivity to be 100%, in a high-risk population (370 of 687 patients underwent US). Our study is the only report to unitize MRI, US, and MMG before PM. However, occult cancers were found in 11.3% of all removed breasts at the time of PM, which indicates that the sensitivity of MRI + MMG + US is not 100% as reported by Kuhl et al. There seem to be limitations of combination surveillance modalities including MRI for patients with *BRCA* mutations.

### BRCA1/2^MUT+^ rates

*BRCA* mutation rates and occult cancer rates do not seem to be related in the studies cited in Table 9. For example, in the study of Burger et al. [8] on women who underwent PM (*n* = 83 in 71 patients) and SLNB (*n* = 1522 in 1498 patients) between 2005 and 2010 in UK, the rate of *BRCA* mutation in the 71 patients was 8.5% and the occult cancer rate was 4.8%, which is similar to 5% reported by van Sprundel et al. [10] among a 100% *BRCA1*/*2*^*MUT*+^ population. Kauff et al. [14] compared prevalence of histopathologic lesions in PM (performed between 1987 and 2001 in USA) specimens from women with *BRCA* mutations and in age and race-matched cadaver mastectomy specimens and found that high-risk epithelial proliferative lesions (including DCIS) are more common in the unaffected breasts of women with known *BRCA* mutations than in women of the comparison group. However, they said that determining whether these lesions are more common in women with *BRCA* mutations than in those without will require direct comparison to women without mutations or with low risk for carrying mutations.

### Pathological methods

Pathological examination methods vary among the papers cited in Table 9. Some studies evaluated 2–4 sections per quadrant of the breast and a section of the nipple, and another evaluated them by 5 mm slices and radiological examination; the methods of the others are not known. Boughey et al. [9] examined specimens (from PMs, between 2000 and 2005, USA) of at least 2 sections per each quadrant and nipple; the specimens were also macroscopically sliced and any areas found abnormal by palpation were evaluated further at the pathologists’ discretion. They noted a 5% occult cancer rate, including 2 IDCs. In the study of Hoogerbrugge et al. [13], the specimens (from PM between 1989 and 2001, Netherlands) were cooled and sliced in serial sections with approximately 5 mm intervals. Radiographs were made from the tissue slices. Suspicious lesions and randomly selected areas from each quadrant and the nipple were sampled, with a mean number of 19 samples per specimen. With this method, they detected 9.3% occult cancer including one 4 mm IDC, and the occult cancer rate would have been higher than 9.3% if LCIS were counted. In the other studies, more detailed pathological examinations might have detected higher occult cancer rates. In the current study, we divided PM specimens about 1 cm serially and then, examined their entirety, and noted 11.3% occult cancer, including a 5 mm IDC. At present, there is not a strict histopathological protocol for PM specimens. However, there exist pathological guidelines for ovarian cancer [23]. Similarly, standardized guidelines for examining PM specimens may be required, as we might easily have missed the aforementioned-5-mm occult cancer. In addition to this, occult cancer should be defined in the protocol, for example, as to whether LCIS can be included in the occult cancer.

This investigation is limited by the fact that it is a registration study from seven institutions and may not fairly reflect the entire population of Japanese *BRCA* mutation carriers.

## Conclusions

Our report showed a relatively higher incidence rate of occult cancer at 11.3% in PM specimens despite thorough pre-operative radiological evaluations, which included a breast MRI. Considering the occult cancer rates and the various pathological methods of our study and published studies, we propose the necessity of a histopathological protocol.
